# Authors’ Reply: Ambiguity in Statistical Analysis Methods and Nonconformity With Prespecified Commitment to Data Sharing in a Cluster Randomized Controlled Trial

**DOI:** 10.2196/57422

**Published:** 2024-04-03

**Authors:** Diego Arguello

**Affiliations:** 1 Human Performance and Exercise Science Lab Department of Health Sciences Northeastern University Boston, MA United States

**Keywords:** prolonged sedentary behavior, sedentary behavior, sit-to-stand desks, treadmill desks, physical activity promotion, workplace wellness, seated office workers, move more and sit less

We appreciate the thoughtful commentary [1] on our study [2] and address the raised concerns.

One pertains to perceived ambiguity in reporting statistical methodology, presumably prompting inquiry into whether analyses accounted for clustering and nesting. We clarify that our choice of a cluster randomized controlled trial design was driven by practical implications (eg, space modification and experimental contamination)—valid reasons for the study design. While this determined the study design, we were interested in the participant-average treatment effect. We feel this is affirmed in our aims and note the perceived ambiguity and level of detail requested by the letter writers were not raised during multiple reviews, and this response to the letter further clarifies our analytical approach.

We agree that type I error inflation is a concern if clustering is not addressed in the study design phase or during analyses. We also recognize that this is an active area of study, and work is ongoing to determine optimal approaches for small-sample study designs [3]. Importantly, the current recommendation is to develop the analytical plan based on goals of the analyses (ie, exploratory outcomes in this study) and various study-specific characteristics (selected examples for our study: random cluster size independent of other data and wide physical distribution of within-cluster participants) [3]. Due to such study-specific considerations, clusters were deemed to be noninformative [4], and our approach—“random-intercept mixed linear models that accounted for repeated measures and clustering effects” [2]—included a random effect for clusters to model any potential correlational structure and interparticipant dependency within clusters [5]. Additionally, we used the Kenward-Rogers method to preserve nominal type I error. The method adjusts for *df* to account for hierarchical complexity of data, including potential nesting and variable or small cluster sizes [3]. We also acknowledged uneven cluster size as a study limitation [2].

Second, while we clearly conveyed the exploratory nature of the analyses aimed at developing hypotheses, we were also conservative by avoiding confirmatory conclusions based on type I error rates [2]. While it is known that conservative analyses may be counterproductive for exploration [6], our careful approach—relying on sound, disparate methods sufficiently accounting for any potential within-cluster dependence and variable cluster size, along with a cautious interpretation strategy—was appropriate for the study objective.

Regarding data sharing, we have previously shared other data and received data from the community to advance multiple areas of inquiry. However, we primarily rejected this request and a competing industry request, given ongoing small business and financial interests leveraging this and the associated body of work; we communicated this to the journal (August 2023) after the request in question was made. Such considerations are necessary to avoid setting a precedent and in the context of any potential for these interests to be compromised. An example of such considerations includes federal funding agencies supporting small business innovation research, allowing awardees to withhold related data to protect endeavors similar to ours. Implying that findings are untrustworthy due to such considerations would incorrectly render a substantial body of such work as the same.
